# Gut microbiota mediate early life stress-induced social dysfunction and anxiety-like behaviors by impairing amino acid transport at the gut

**DOI:** 10.1080/19490976.2024.2401939

**Published:** 2024-09-11

**Authors:** Jiushuang Zhu, Zhuoting Zhong, Lijie Shi, Ling Huang, Chunqiao Lin, Yan He, Xiuwen Xia, Tiane Zhang, Weijun Ding, Youjun Yang

**Affiliations:** School of Basic Medical Sciences, Chengdu University of Traditional Chinese Medicine, Chengdu, P. R. China

**Keywords:** Early life stress, gut microbiota, neurobehavioral abnormalities, synaptic transmission, medial prefrontal cortex

## Abstract

Early life stress alters gut microbiota and increases the risk of neuropsychiatric disorders, including social deficits and anxiety, in the host. However, the role of gut commensal bacteria in early life stress-induced neurobehavioral abnormalities remains unclear. Using the maternally separated (MS) mice, our research has unveiled a novel aspect of this complex relationship. We discovered that the reduced levels of amino acid transporters in the intestine of MS mice led to low glutamine (Gln) levels in the blood and synaptic dysfunction in the medial prefrontal cortex (mPFC). Abnormally low blood Gln levels limit the brain’s availability of Gln, which is required for presynaptic glutamate (Glu) and γ-aminobutyric acid (GABA) replenishment. Furthermore, MS resulted in gut microbiota dysbiosis characterized by a reduction in the relative abundance of *Lactobacillus reuteri* (*L. reuteri*). Notably, supplementation with *L. reuteri* ameliorates neurobehavioral abnormalities in MS mice by increasing intestinal amino acid transport and restoring synaptic transmission in the mPFC. In conclusion, our findings on the role of *L. reuteri* in regulating intestinal amino acid transport and buffering early life stress-induced behavioral abnormalities provide a novel insight into the microbiota-gut-brain signaling basis for emotional behaviors.

## Graphical abstract

**Figure:**
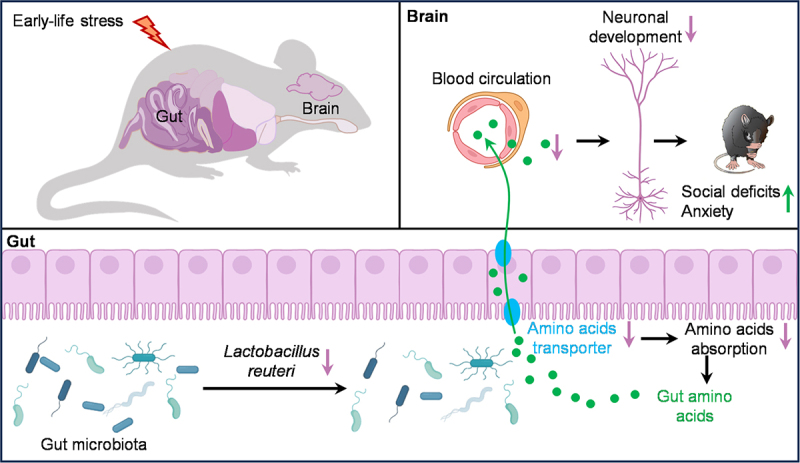

## Introduction

Childhood traumatic experiences have been associated with the development of neuropsychiatric disorders.^1–3^ Studies in humans and animals have shown that maternal separation (MS) is a critical stressor in early life and can lead to social dysfunction and anxiety in individuals and animals.^4–8^ Gut microbiota plays an essential role in the bidirectional communication between the gut and the brain, known as the microbiota-gut-brain axis.^9–11^ Gut microbiota is thought to be a modulator of the host’s behaviors, including social behavior and anxiety, through complex interactions with the host’s central nervous system (CNS).^12,13^ Early life stress, including MS, has been reported to alter the gut microbiota.^14,15^ Moreover, consumption of a multi-strain probiotic mixture could alleviate anxiety and depression symptoms in MS mice,^16^ suggesting gut microbial dysbiosis may be a key mediator in MS-induced neuropsychiatric symptoms.

The brain depends on a constant supply of amino acids from the periphery to maintain proper physiological functions. The impairment of amino acid transportation into the brain causes autism-like phenotypes in mice.^17^ The intestine absorbs amino acids from dietary food through amino acid transporters on the membrane of the intestinal mucosa.^18^ These absorbed amino acids can react with alpha-ketoglutaric acid (α-KG) under the catalyst of aminotransferase in the liver and other organs to form glutamate (Glu), which can not cross the blood-brain barrier.^19^ However, Glu can react with ammonia to form glutamine (Gln) under the catalyst of Gln synthetase. Unlike Glu, Gln can cross the blood-brain barrier and enter the brain.^19^ Gln plays a multifaceted role in neuronal function, including replenishing presynaptic Glu and γ-aminobutyric acid (GABA),^20,21^ two predominant neurotransmitters in the CNS. Gln deficiency in the brain has been implicated in the pathophysiology of neurological diseases such as Alzheimer’s disease,^22^ temporal lobe epilepsy,^23^ Parkinson’s disease,^24^ and major depressive disorder (MDD).^25,26^ Recently, researchers discovered that the gut microbiota could regulate the Glu levels in the brain by modulating the expression of intestinal amino acid transporters in a mouse model of autism.^27^ Here, our investigation delves into the potential role of amino acids as microbiota-gut-brain signaling molecules that connect gut microbial metabolites to early life stress vulnerability in the host.

## Results

### MS mice exhibit behavioral abnormalities and gut microbiota dysbiosis

**Figure 1.**
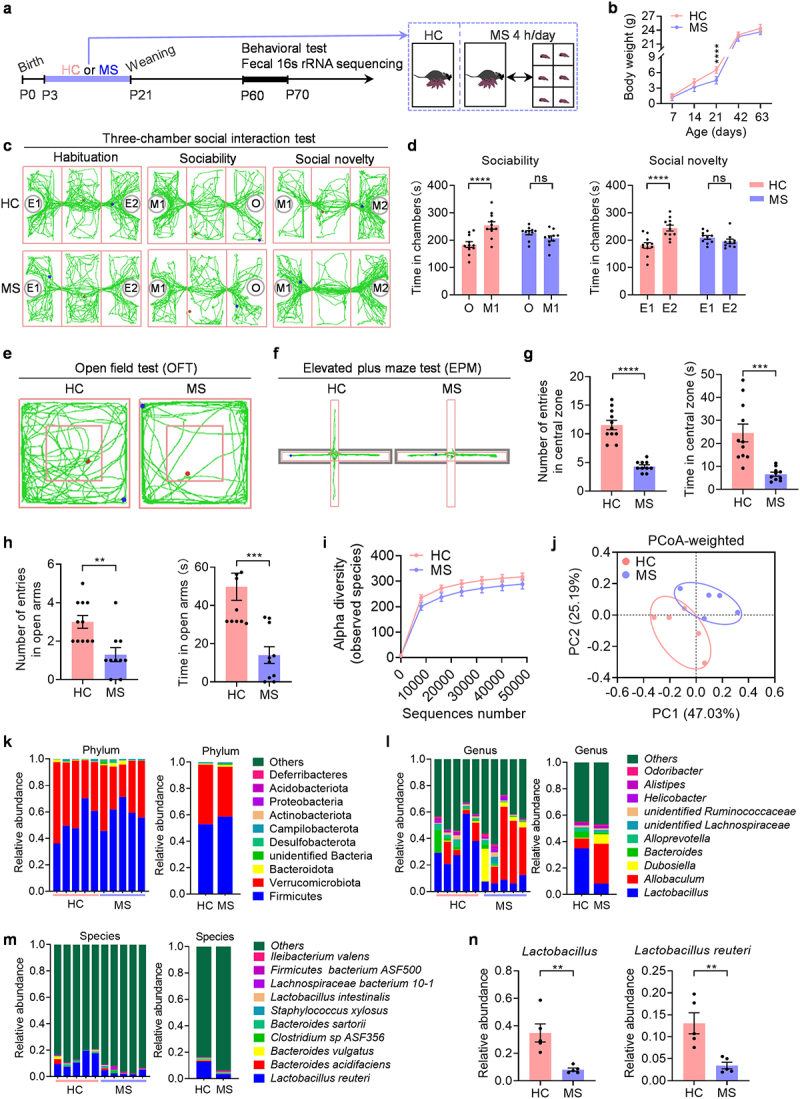
Behavioral dysfunction and dysbiosis of the gut microbiota in MS mice.

We examined the long-term effects of early life stress on behaviors by subjecting mice to the MS protocol to induce early life stress (Figure 1(a)). Initially, MS caused a decrease in weight gain at weaning (P21), followed by a return to normal weight levels in adulthood (Figure 1(b)). Subsequently, we conducted behavioral tests to assess the impact of MS on social ability and anxiety. The three-chamber social interaction test was used to evaluate sociability and social novelty (Figure 1(c)). During the habituation phase, neither MS mice nor HC controls exhibited a preference for the two empty cages placed in the right and left chambers (Supplementary Figure S1a). In the sociability test, HC mice showed a significantly higher preference for an unfamiliar mouse (M1) over an inanimate object (O) (Figure 1(d)). Conversely, MS mice did not show a significant difference in the time spent with M1 and O (Figure 1(d)). Further analysis of social novelty revealed that HC mice spent more time interacting with a new unfamiliar mouse (M2) than with a previously encountered familiar mouse (M1) (Figure 1(d)). In contrast, MS mice did not show a significant difference in the time spent with M2 and M1 (Figure 1(d)). In summary, these results indicate that MS mice display impaired social communication.

(a) The experimental timeline for the MS procedure. (b) The body weight of HC mice and MS mice. (c) Representative traces of HC and MS mice in three-chamber social interaction test. (d) The resident time in chambers of the test mice in the sociability and social novelty tests (*n* = 10-11 mice from different dams for each group). (e) Representative traces of HC and MS mice in the open field test. (f) Representative traces of HC and MS mice in the elevated plus maze test. (g) The number of entries into the center and the time spent in the central zone in the open field test (*n* = 10-11 mice from different dams for each group). (h) The number of entries into the open arms and the time spent in the open arms in elevated plus maze test (*n* = 10-11 mice from different dams for each group). (i) α diversity of fecal 16S rRNA sequencing data from HC and MS mice (*n* = 5 mice from different dams for each group). (j) PCoA of weighted UniFrac distances from the averaged rarefied 16S rRNA gene dataset (*n* = 5 mice from different dams for each group). (k) Taxonomic distributions of bacteria from fecal 16S rRNA sequencing data at phylum levels (*n* = 5 mice from different dams for each group). (l) Relative abundances of the top 10 bacterial genus from fecal 16S rRNA sequencing data (*n* = 5 mice from different dams for each group). (m) Relative abundances of the top 10 bacterial species from fecal 16S rRNA sequencing data (*n* = 5 mice from different dams). (n) Relative abundances of *Lactobacillus* and *L. reuteri* (*n* = 5 mice from different dams for each group). Data were shown as mean ± SEM. *P < 0.05, **P < 0.01, ***P < 0.001, ****P < 0.0001; ns, no significant difference. Statistical differences were determined by two-tailed unpaired Student’s *t*-test (g, h, n) and two-way ANOVA with Sidak’s multiple-comparison test (b, d). Statistical details are provided in Supplementary Table S1.

To investigate anxiety-like behaviors, we conducted the open field test (OFT) (Figure 1(e)) and the elevated plus maze (EPM) test (Figure 1(f)). In the OFT, mice exposed to maternal separation (MS) showed reduced entries into the center, spent less time in the center zone, and traveled shorter distances within the center area compared to the control group (HC) (Figure 1(g) and Supplementary Figure S1b). Similarly, in the EPM test, MS mice displayed fewer entries into the open arms, spent less time in the open arms (Figure 1(h)), and traveled reduced distances in the open arms compared to the control group (Supplementary Figure S1c). These results indicate that MS leads to increased anxiety. Notably, the observed social deficits and heightened anxiety in MS mice were not due to changes in movement, as the speed of movement during the behavioral tests was the same for both groups (Supplementary Figure S1d-f).

The gut microbiota has been found to be influenced by MS^14,16^ and disturbances in gut microbiome are strongly associated with social deficits and anxiety.^13,16,28–30^ To determine whether MS altered the gut microbiota, we examined fecal microbiota composition by 16S ribosomal RNA (rRNA) sequencing. First, we assessed the α diversity by comparing the observed species. We found a decrease in microbial diversity in MS mice compared to HC controls (Figure 1(i)), indicating a loss of specific bacterial taxa in MS mice. Subsequently, we evaluated β diversity by Principal coordinates analysis (PCoA). We found a significant difference in the microbial taxon distribution between MS mice and HC controls (Figure 1(j)), reflecting the variability in microbial communities between the two groups. These results were further supported by taxa summary analysis at bacterial phyla, genera and species levels (Figure 1(k-m)). Notably, MS mice showed a reduction in the relative abundance of *L. reuteri* (Figure 1(n) and Supplementary Figure S1g, h), which can shape host behaviors, especially social behaviors.^12,31–34^ These findings reveal that the composition of the gut microbiota in mice is substantially altered by MS, leading to speculation that the absence of specific gut microbiota, such as *L. reuteri*, may be responsible for the behavioral abnormalities in MS mice.

### The gut microbiota is necessary and sufficient for ms-induced behavior abnormalities

To investigate the role of gut microbiota in social deficits and anxiety caused by maternal separation (MS), we conducted a study to see if housing MS mice with HC controls could prevent the development of social deficits and anxiety. At P21, we placed one MS mouse in a shared living environment with three HC mice (Supplementary Figure S2a). We found that this cohousing could reverse the microbiota changes observed in MS mice, bringing their bacterial profiles more in line with those of co-housed HC controls (Supplementary Figure S2b, c). Cohousing could reverse the microbiota changes observed in MS mice, bringing their bacterial profiles more in line with those of co-housed HC controls. Next, we wondered whether cohousing would rescue social deficits and anxiety in MS mice. While cohousing improved sociability, it failed to rescue preference for social novelty (Supplementary Figure S2d, e). We next examined whether cohousing can contribute to the regulation of anxiety-related behaviors. In the OFT, cohousing had no impact on the behavior of the co-housed MS mice (Supplementary Figure S2f, g). However, co-housed MS mice showed more exploration in the open arms of the EPM compared to MS mice (Supplementary Figure S2h,i). These findings indicate that co-housing with HC mice can partially alleviate the social deficits and anxiety-like behaviors in MS mice.

To further confirm the relationship between gut microbiota and MS-induced social deficits and anxiety, we performed fecal microbiota transplantation (FMT) from HC and MS-exposed mice into antibiotics (Abx)-pretreated mice (HC-FMT and MS-FMT, respectively, Supplementary Figure S3a). Results showed that these two groups’ α diversity was similar (Supplementary Figure S3b). However, the β diversity showed a significant difference in the microbial taxon distribution between HC-FMT mice and MS-FMT mice (Supplementary Figure S3c). As expected, the HC-FMT-recipient mice showed normal social and anxious behaviors (Supplementary Figure S3d, e). In contrast, the recipient mice that received fecal microbiota from MS donor mice remained socially impaired and showed increased anxiety (Supplementary Figure S3f-i). These data indicate that MS-altered microbes are sufficient for behavioral abnormalities in the host.

To directly determine whether the gut microbiota influences social and anxious behaviors, we depleted the gut microbiota of HC mice with long-term antibiotics treatment (drinking water, P21- P63) (Supplementary Figure S4a). 16S rRNA gene sequencing showed that antibiotic treatment decreased the α diversity of gut microbiota (Supplementary Figure S4b). β diversity analysis also showed a marked difference in the gut microbiota composition between antibiotics-treated and control mice (Supplementary Figure S4c). These results suggest that antibiotics effectively eliminate gut microbiota. Consistent with previous studies in germ-free mice,^12,30,35^ the antibiotics-treated mice exhibit social deficits (Supplementary Figure S4d, e). Although previous studies reported anxiety-like behavior was lower in germ-free mice than in their SPF counterparts,^30,36^ we found that antibiotics treatment increased anxiety (Supplementary Figure S4f-i). These results indicate that the gut microbiota is required for normal social and anxious behaviors.

**Figure 2.**
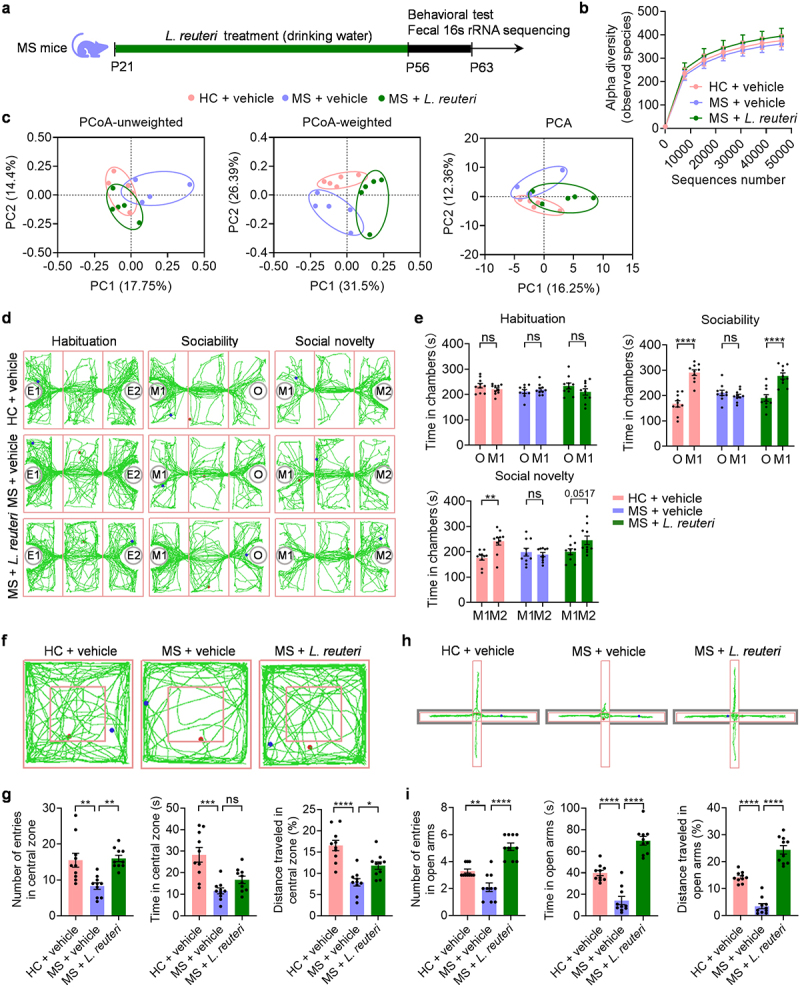
Treatment with *L. reuteri* restores social deficits and anxiety-like behaviors in MS mice.

We identified that the relative abundance of *L. reuteri* was drastically reduced in MS mice compared to HC controls. Studies have found that *L. reuteri* deficiency could lead to social impairment and anxious behaviors, and selective supplementation with *L. reuteri* is efficacious in improving social deficits and anxiety.^12,31^ Thus, we hypothesized that the selective decrease in *L. reuteri* in the gut was causally related to social deficits and increased anxiety in MS mice. To test this hypothesis, we introduced *L. reuteri* into the drinking water of MS mice at weaning for five weeks, after which behavior was investigated (Figure 2(a)). *L. reuteri* treatment increased the α diversity of the gut microbiota but was not significant (Figure 2(b)). However, the β diversity analysis showed significant differences between the *L. reuteri*-treated mice and the vehicle-treated controls (Figure 2(c)). These results imply that *L. reuteri* alters the composition of the gut microbiota of MS mice. Remarkably, treatment with *L. reuteri* improved sociability and preference for social novelty (Figure 2(d,e)). In the open field test, *L. reuteri*-treated MS mice showed increased entries into the central zone and traveled a greater distance in the central zone than vehicle-treated MS mice. (Figure 2(f,g)). The time spent in the center was similar between these two groups (Figure 2(g)). In the EPM test, the MS mice treated with *L. reuteri* were more likely to explore the open arms compared to the MS mice treated with vehicle (Figure 2(h,i)), indicating decreased anxiety. Overall, these data demonstrate that commensal microbe-mediated MS-induced social defects and anxiety and selective microbial intervention with *L. reuteri* rescue social dysfunctions and anxiety-like behaviors in MS mice.

(a) Schematic of the *L. reuteri* treatment. (b) α diversity of the gut microbiota of HC mice, MS mice, and MS + *L. reuteri* mice. (c) PCoA of unweighted UniFrac distances (left), PCoA of weighted UniFrac distances (middle) and PCA plot (right) based on the 16S rRNA gene sequencing dataset from the feces of HC mice, MS mice and MS + *L. reuteri* mice (*n* = 5 mice from different dams for each group). (d) Representative traces of HC mice, MS mice and MS + *L. reuteri* mice in three-chamber social interaction test. (e) The resident time in chambers of the test mice in the three-chamber social interaction test (*n* = 10 mice from different dams for each group). (f) Representative traces of the test mice in the open field test. (g) The number of entries into the center (left), the time spent in the center (middle) and the distance traveled in the central zone (right) of the test mice in open field test (*n* = 10 mice from different dams for each group). (h) Representative traces of test mice in the elevated plus maze test. (i) The number of entries into the open arms (left), the time spent in the open arms (middle) and the distance traveled in the open arms (right) of the test mice in elevated plus maze test (*n* = 10 mice from different dams for each group). Data were shown as mean ± SEM. *P < 0.05, **P < 0.01, ****P < 0.0001; ns, no significant difference. Statistical differences were determined by one-way ANOVA with Tukey’s multiple-comparison test (g, i) and two-way ANOVA with Sidak’s multiple-comparison test (b, e). Statistical details are provided in Supplementary Table S1.

### L. reuteri ameliorates ms-induced neurotransmission dysfunctions

**Figure 3.**
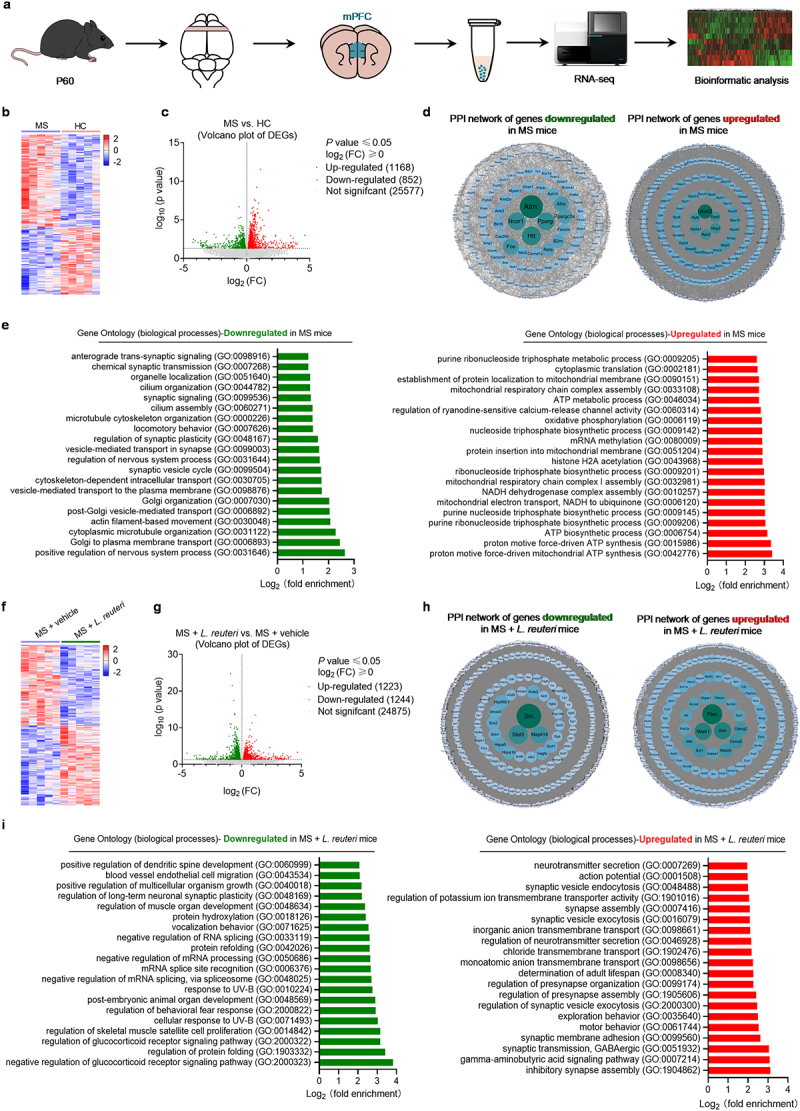
Uncovering potential downstream neuron modulatory mechanisms of *L. reuteri* treatments.

To explore potential downstream mechanisms by which MS may induce social deficits and anxiety, we conducted bulk RNA-sequencing (RNA-seq) of mPFC samples collected from HC mice and MS mice (Figure 3(a)). We focused on the mPFC because of its involvement in social behavior and mood modulation,^37,38^ and studies show that MS can alter neuronal development and functions in this brain region.^39–41^ MS altered the expression of 2020 genes in the mPFC (Figure 3(b,c)). Search tool for the retrieval of interacting genes/proteins (STRING) network analysis of these differentially expressed genes (DEGs) revealed that the top interaction proteins included the upregulation of *Atm*, *Ncor1*, *Pparg*, *Htt*, and *Fos*, and the downregulation of *Uba52*, *Rpl8*, *Rps5*, *Atp5a1*, *Nhp2*, and *Rps3* (Figure 3(d)). The Gene Ontology (GO) enrichment analysis for the biological processes of the downregulated genes in the mPFC of MS mice showed significant enrichment in pathways related to the positive regulation of nervous system processes, intracellular transport, synaptic plasticity, and synaptic transmission (Figure 3(e)). The upregulated genes in the mPFC of MS mice are mainly enriched in pathways related to ATP biosynthetic process and mRNA methylation (Figure 3(e)). These results suggest that MS alters the prefrontal cortical transcriptomic profile and impairs synaptic transmission in this brain region.

(a) Schematic of RNA-seq for mPFC samples from PBS offspring and MIA offspring at P60. (b) Heatmap of differentially expressed genes (DEGs) in the mPFC of MS mice compared with that of HC mice (*n* = 5 mice from different dams for each group). (c) Volcano plot of mPFC transcripts from HC and MS mice (*n* = 5 mice from different dams for each group). (d) Protein interaction network of DEGs with STRING network analyses. (e) Gene Ontology (GO, Biological Process) analysis of differentially expressed genes (DEGs) in the mPFC of MS mice compared with that of HC mice. (f) Heatmap of differentially expressed genes (DEGs) in the mPFC of *L.reuteri*-treated MS mice compared with vehicle-treated MS mice (*n* = 5 mice from different dams for each group). (g) Volcano plot of mPFC transcripts from *L.reuteri*- and vehicle-treated MS mice (*n* = 5 mice from different dams for each group). (h) Protein interaction network of DEGs with STRING network analyses. (i) Gene Ontology (GO, Biological Process) analysis of differentially expressed genes (DEGs) in the mPFC of *L. reuteri*-treated MS mice compared with that of vehicle-treated MS mice.

To gain insight into how colonization with *L. reuteri* may alter brain function to alleviate social deficits and anxiety in MS mice, we analyzed the transcriptomic profiling from the mPFC of *L. reuteri*- and vehicle-treated MS mice. Our data show that *L. reuteri* treatment significantly changes the gene expression profile in the mPFC (Figure 3(f,g)). STRING revealed that the top interaction proteins included upregulation of Src, Stat3, and Mapk14, and downregulation of Pten, Mapk1, and Atm (Figure 3(h)). GO enrichment analysis for the biological processes of the downregulated genes in the mPFC of *L. reuteri*-treated MS mice revealed significant enrichment in the glucocorticoid receptor signaling pathway and mRNA processing pathways (Figure 3(i)). The upregulated genes in the mPFC of *L. reuteri*-treated MS mice are mainly enriched in pathways related to synapse assembly and GABAergic synaptic transmission (Figure 3(i)). The findings indicate that MS causes changes in gene expression in the frontal cortex compared to HC controls, including reduced gene expression in synaptic development and transmission. Interestingly, treatment with *L. reuteri* has shown promise in restoring the expression of genes related to synaptic transmission in the mPFC of mice with MS. This discovery is intriguing because it suggests that the beneficial effects of *L. reuteri* treatment on abnormal behaviors in mice with MS may be due to the improvement of neurotransmission dysfunction caused by MS.

**Figure 4.**
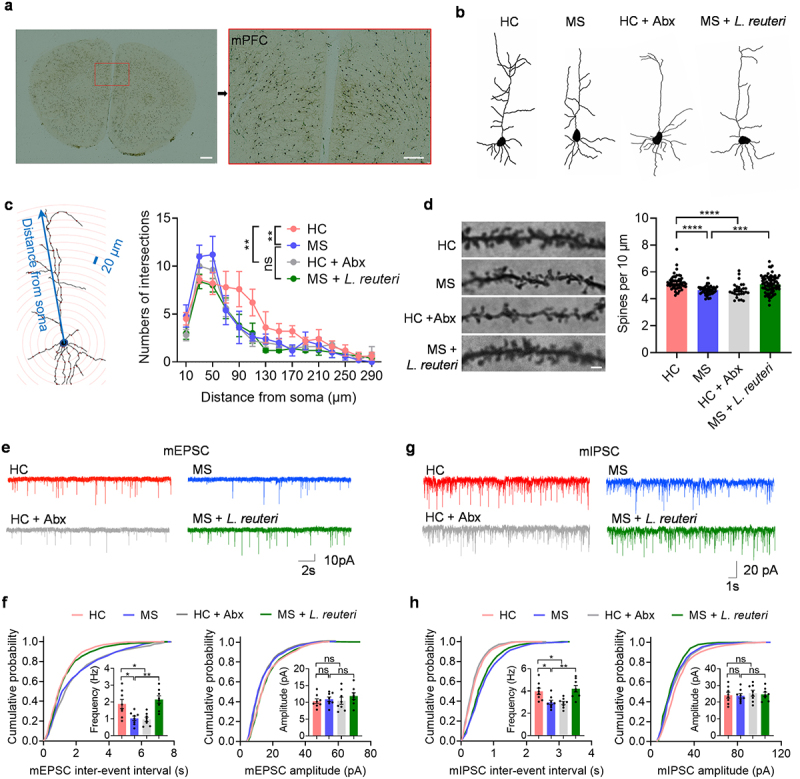
*L. reuteri* rescues Glutamatergic and GABAergic transmission in the mPFC of MS mice.

Structural and functional abnormalities in neurons or dendritic spines could lead to neurotransmission dysfunction.^42–44^ Long-term exposure to stressful experiences can cause structural changes in neurons.^45^ Stressed animals may show dendritic retraction and loss of dendritic spines in brain regions like the hippocampus, sensory cortex, and PFC.^45–47^ The PFC, essential for higher-order thinking, is particularly sensitive to stress compared to other brain regions.^48,49^ Even briefly exposing a stressful stimulus can lead to dendritic retraction in the PFC.^50^ To study whether MS alters the structure of neurons and the number of dendritic spines in the mPFC, we performed Golgi staining and reconstructed neuronal structures (Figure 4(a,b)). Sholl analysis showed that MS reduced dendritic complexity and dendritic spine density in mPFC layer II/III pyramidal neurons (Figure 4(c,d)). Interestingly, antibiotic treatment also induced neuronal structural changes similar to those in MS mice (Figure 4(c,d)). *L. reuteri* treatment does not restore the structural complexity of mPFC pyramidal neurons (Figure 4(c)). However, the numbers of spines on dendrites of mPFC pyramidal neurons were restored by *L. reuteri* treatment (Figure 4(d)). Overall, these results indicate that maternal separation (MS) reduced the complexity of dendrites and the density of spines in the pyramidal neurons of the medial prefrontal cortex (mPFC) in mice. Furthermore, the findings suggest that the gut microbiota plays a crucial role in supporting neuronal development.

(a, b) Representative picture of Golgi-stained projection neuron in the mPFC (a) and its reconstructed structures (b). (c) Sholl analysis examples of the reconstructed structures in each group (HC, *n* = 55 cells from 5 mice; MS, *n* = 46 cells from five mice; HC +Abx, *n* = 28 cells from five mice; MS + *L. reuteri*, *n* = 77 cells from five mice). (d) Representative confocal images of Golgi-stained apical dendrites (left) and the average number of dendritic spines/10 μm of apical dendrite from each group (right). Scale bar: 5 μm. (e) Representative mEPSC traces from each group. (f) Cumulative distribution of mEPSC interevent intervals (left) and amplitude (right). Inset, the histogram of frequency and amplitude of mEPSC from each group (*n* = 8 cells from 5 mice for each group). (g) Representative mIPSC traces from each group. (h) Cumulative distribution of mIPSC interevent intervals (left) and amplitude (right). Inset is the histogram of frequency and amplitude of mIPSC for each group. (*n* = 8 cells from 5 mice for each group). Data were shown as mean ± SEM. *P < 0.05, **P < 0.01, ****P < 0.0001; ns, no significant difference. Two-way ANOVA determined statistical differences with Tukey’s multiple-comparison test (c) and one-way ANOVA with Tukey’s multiple-comparison test (d, f, h). Statistical details are provided in Supplementary Table S1.

Next, we characterized the changes in the neuronal properties, including miniature excitatory postsynaptic currents (mEPSCs) and miniature inhibitory postsynaptic currents (mIPSCs), in this brain region. In line with reduced spines, the mEPSC frequency of MS mice and antibiotics-treated mice was decreased, while the amplitude remained unchanged. (Figure 4(e,f)). In addition, we found that the frequency of mIPSCs was reduced in mice with MS and antibiotic-treated mice, but not the amplitude, compared with neurons from healthy control mice (Figure 4(g,h)). Then, we examined whether *L. reuteri* treatment could reverse synaptic dysfunction in the mPFC of MS mice. Recordings from mPFC pyramidal neurons demonstrated that MS decreased the frequency of mEPSCs and mIPSCs, an effect that was reversed by *L. reuteri* (Figure 4(e-h)). These results show that both Glutamatergic and GABAergic transmissions are altered by MS or antibiotic treatment, suggesting that gut microbiota may mediate synaptic transmission abnormalities in MS mice. Supplementation with *L. reuteri* can effectively rescue MS-induced Glutamatergic and GABAergic deficits in the mPFC.

### L. reuteri improves the Gln- Glu/gaba cycle

**Figure 5.**
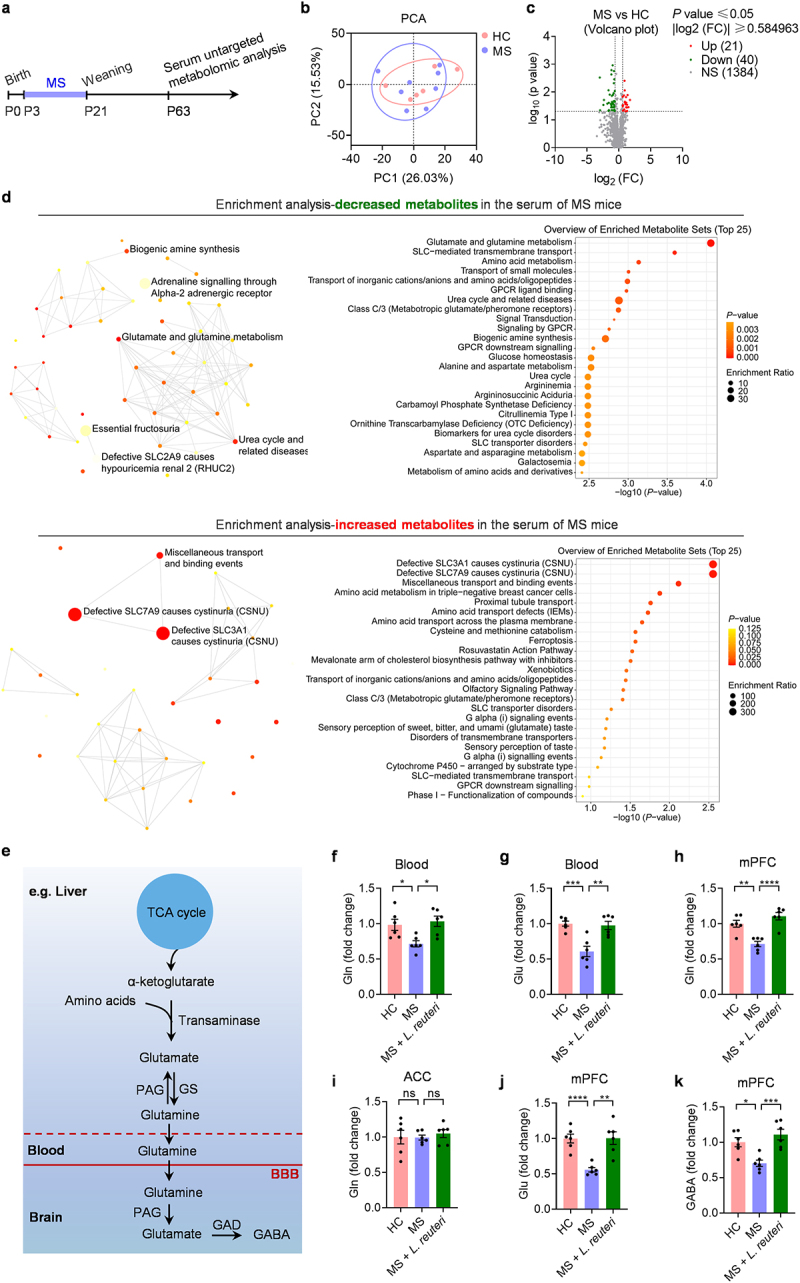
Impairment of the Gln-Glu/GABA cycle in the mPFC mediates social deficits and anxiety.

Since the gut microbiota is distant from the brain and influences the availability of various biochemicals in the bloodstream, we suggested that microbial-regulated metabolites could potentially affect the susceptibility to social deficits and anxiety in MS mice by translocating to the CNS. To investigate this, we profiled biochemicals in the blood of HC mice and MS mice (Figure 5(a)). The serum metabolomic profiles from HC mice were similar to those from MS mice (Figure 5(b)). However, out of 1445 serum metabolites identified in HC and MS mice, 61 metabolites were significantly altered by MS. Among these, 21 were increased, and 40 were decreased (Figure 5(c)). Enrichment analysis of differential metabolites showed that MS decreased amino acid-related metabolism in the blood, especially the reduced Glu and Gln metabolism (Figure 5(d)). Furthermore, MS increased the metabolites related to cystinuria, which is caused by defective in amino acid transporter, such as SLC7A9 and SLC3A1 (Figure 5(d)). These findings reveal that the MS regulates amino acid-related metabolites in the blood of mice.

(a) Schematic of serum metabolomes analysis experiment for HC mice and MS mice. (b) Principal component analysis (PCA) of serum metabolomes from HC and MS mice on P63 (*n* = 7-8 mice from different dams for each group). (c) Volcano plot of serum metabolites from HC and MS mice. (d) Top 25 enriched pathways for the decreased and increased serum metabolites in MS mice compared with HC mice. (e) Schematic diagram of peripheral glutamine crossing the blood-brain barrier and converting to glutamate and GABA. (f-k) The effect of *L. reuteri* treatment on Gln, Glu and GABA levels in the blood and specific brain regions (*n* = 6 mice from different dams for each group). Data were shown as mean ± SEM. *P < 0.05, **P < 0.01, ***P < 0.001, ****P < 0.0001. Statistical differences were determined by one-way ANOVA with Tukey’s multiple-comparison test (f-k). Statistical details are provided in Supplementary Table S1.

Some amino acids can act as excitatory or inhibitory neurotransmitters or as neurotransmitter precursors and thus affect glutamatergic and GABAergic transmission. Among the amino acids, glutamate (Glu) was focused on because it is the major excitatory neurotransmitter in the brain, and its related metabolites showed the most significant reduction in the blood of mice with multiple sclerosis (MS). In addition, Glu can be decarboxylated to produce GABA, the brain’s predominant inhibitory neurotransmitter. Since Glu cannot pass through the blood-brain barrier (BBB), it is synthesized in the brain by the conversion of other substances, or it can be reversibly generated from Gln, which can pass through the BBB (Figure 5(e)). Therefore, changes in brain Glu levels may be due to the conversion of other substances in the brain, as well as the transport of serum Gln to the brain via the BBB. We first focused on factors outside the brain, such as dietary intake and metabolic processes, that can influence the Gln level in the brain. Therefore, we examined the levels of Gln and Glu in the blood using ELISA and found that MS mice showed decreased Gln and Glu levels in the serum. (Figure 5(f,g)), which is consistent with data from untargeted metabolomics, suggesting that the decreased Gln level in the blood might result in a decrease of Gln in the brain. Next, we examined the levels of Gln in the brain by ELISA and found that the MS mice displayed a significant reduction in the levels of Gln in the mPFC (Figure 5(h)). We found no detectable differences in the levels of Gln in the ACC, a brain region also involved in social behavior (Figure 5(i)). As Gln is a precursor for the synthesis of both Glu and GABA, we examined the levels of Glu and GABA in the brain by ELISA. We found that MS mice showed a significant decrease in the levels of Glu and GABA in the mPFC compared with HC controls (Figure 5(j,k)). MS mice supplemented with *L. reuteri* restored the levels of Gln and Glu in the blood (Figure 5(f,g)). Moreover, MS mice supplemented with *L. reuteri* had elevated Gln, Glu, and GABA levels in the mPFC (Figure 5(h,j,k)). These results indicate that limited Gln availability may underlie MS-induced neurotransmission dysfunction and behavioral abnormalities and that supplementation with *L. reuteri* increased Gln levels in the blood and brain. This may be the potential mechanism by which *L. reuteri* ameliorates neurotransmission dysfunction and behavioral abnormalities in MS mice.

**Figure 6.**
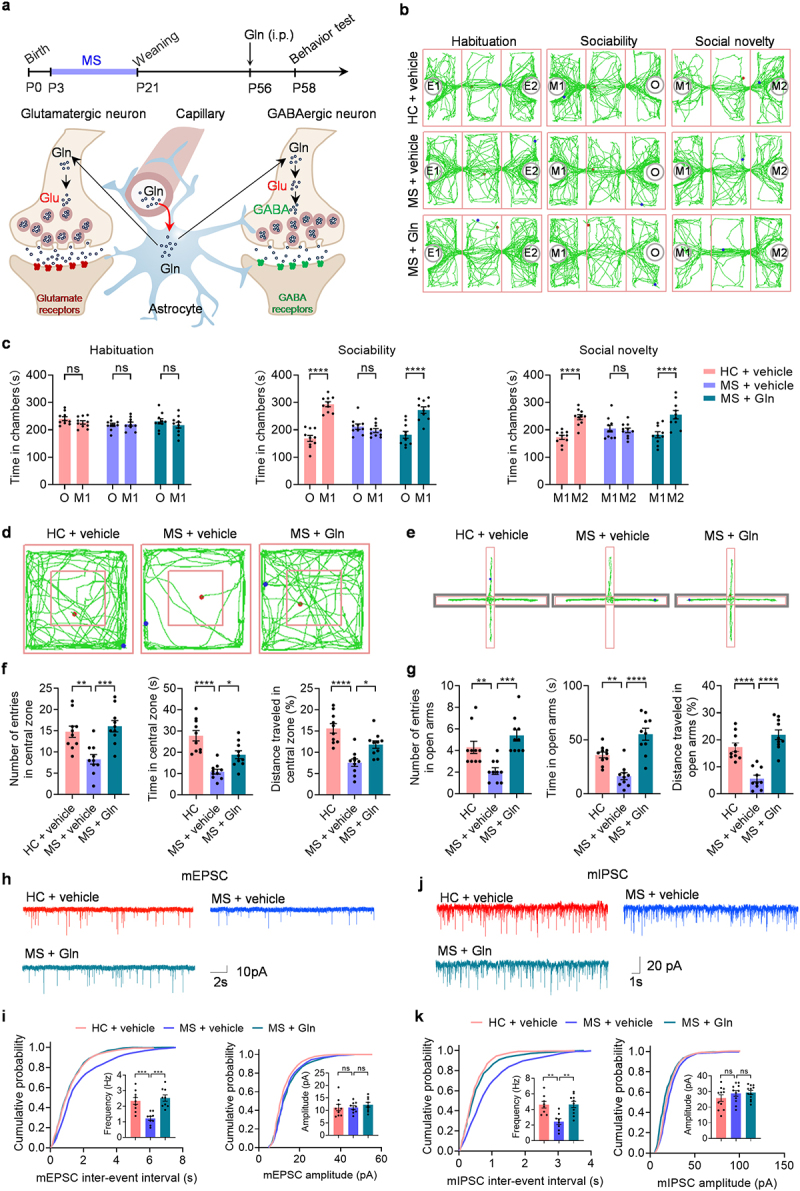
Administration of Gln rapidly reverses behavioral abnormalities and improves neurotransmission in the mPFC of MS mice.

Therefore, we theorized that a shortage of amino acids in the peripheral system could impact behaviors by reducing the availability of Gln. This amino acid can cross the blood-brain barrier and is a precursor for producing two major neurotransmitters, Glu and GABA, in the CNS (Figure 6(a)). We examined whether administering Gln (intraperitoneal injection, 0.25 g per kg) could rescue the behavioral abnormalities in MS mice (Figure 6(a)). Indeed, we found that Gln rescued MS-induced social deficits and anxiety (Figure 6(b-g)), supporting the essential role of Gln in early life stress-coping processes. These results suggest that the limited availability of Gln may be the cause of behavior abnormalities in MS. Additionally, Gln may play a role in mediating the beneficial effects of *L. reuteri* on social deficits and anxiety in MS mice.

(a) Experimental time for Gln administration and schematic of the Gln-Glu/GABA cycle in the brain. (b) Representative traces of HC mice, MS + vehicle mice and MS + Gln mice in three-chamber social interaction test. (c) The resident time in chambers of the test mice in three-chamber social interaction test (*n* = 10 mice from different dams for each group). (d) Representative traces of the test mice in open field test. (e) Representative traces of the test mice in elevated plus maze test. (f) The number of entries into the center (left), the time spent in the center (middle) and the distance traveled in the central zone (right) of the test mice in open field test (*n* = 10 mice from different dams for each group). (g) The number of entries into the open arms (left), the time spent in the open arms (middle) and the distance traveled in the open arms (right) of the test mice in elevated plus maze test (*n* = 10 mice from different dams for each group). (h) Representative mEPSC traces from each group. (i) Cumulative distribution of mEPSC interevent intervals (left) and amplitude (right). Inset, the histogram of frequency and amplitude of mEPSC from each group (*n* = 9 cells from 5 mice for each group). (g) Representative mIPSC traces from each group. (k) Cumulative distribution of mIPSC interevent intervals (left) and amplitude (right). Inset is the histogram of frequency and amplitude of mIPSC for each group. (*n* = 9 cells from 5 mice for each group). Data were shown as mean ± SEM. *P < 0.05, **P < 0.01, ****P < 0.0001; ns, no significant difference. Statistical differences were determined by two-way ANOVA with Tukey’s multiple-comparison test (c) and one-way ANOVA with Tukey’s multiple-comparison test (f, g, i, k). Statistical details are provided in Supplementary Table S1.

Next, we wondered whether Gln treatment would restore Glutamatergic and GABAergic function in the mPFC. Therefore, we recorded mIPSCs and mEPSCs in the mPFC using whole-cell patch-clamp techniques. Our findings showed that supplementing with Gln restores the frequency of mEPSCs and mIPSCs in pyramidal neurons of the mPFC (Figure 6(h-k)). These results suggest that Gln availability may be limited in MS mice, leading to Glutamatergic and GABAergic function impairment. Reactivating the Gln-Glu/GABA cycle can reverse MS-induced Glutamatergic and GABAergic deficits in the mPFC.

### L. reuteri restores intestinal amino acid transporter levels

**Figure 7.**
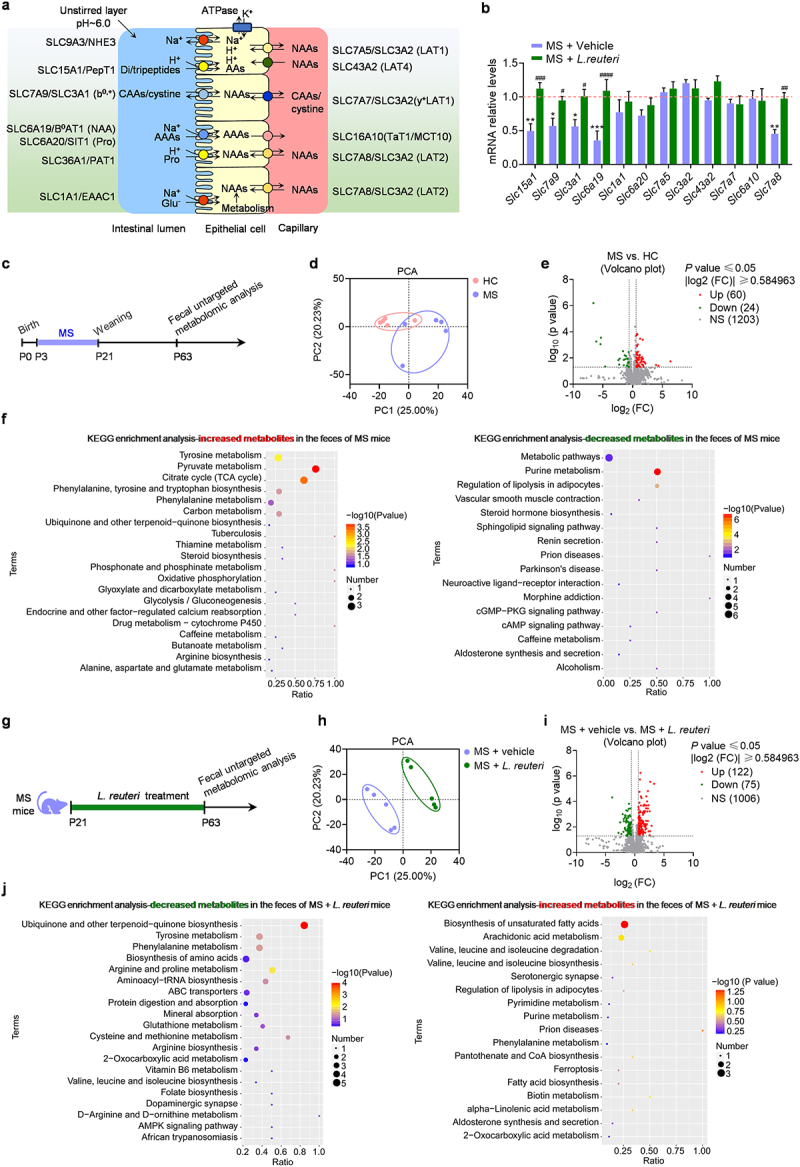
*L. reuteri* restores the expression of intestinal amino acid transporters in MS mice.

We subsequently explored the mechanisms underlying the reduced serum Glu and Gln levels in the MS mice. Intestinal transport of amino acids to the blood is one of the primary sources in the blood (Figure 7(a)). Therefore, we examined the expression of amino acid transporters in the small intestines using quantitative PCR (qPCR) analyses. Among these transporters, the mRNA levels of Di/tripeptides transporter (*Slc15a1*), CAAs/cystine transporters (*Slc7a9*and *Slc3a1*), and neutral amino acid transporters responsible for Gln and aromatic amino acid (phenylalanine, tyrosine, and tryptophan) transporters (*Slc6a19* and *Slc7a8*) were significantly reduced in MS mice (Figure 7(b)). SLC15A1 is the oligopeptide transporter (PepT1) that mediates the uptake of oligopeptides (di- and tripeptides), the major end-products of protein digestion. The defective SLC7A9 and SLC3A1 will lead to cystinuria, characterized by defective cystine and dibasic amino acid transport across the apical membrane of epithelial cells of the small intestine and the renal proximal tubules. These results indicated that the decreased expression of amino acid transporters in intestinal epithelial cells may mediate the reduced serum amino acid levels in MS mice.

(a) The figure shows major amino acid transporters contributing to the absorption of amino acids. (b) qPCR analysis of mRNA levels of amino acid transporters in the small intestine of mice (*n* = 5 mice from different dams for each group). (c) Schematic of fecal metabolomes analysis experiment for HC mice and MS mice. (d) PCA of fecal metabolomes from HC and MS mice on P63 (*n* = 5 mice from different dams for each group). (e) Volcano plot of fecal metabolites for HC and MS mice (*n* = 5 mice from different dams for each group). (f) KEGG enrichment analysis for differential fecal metabolites in MS mice compared with HC mice. (g) Schematic of fecal metabolomes analysis experiment for MS mice after *L. reuteri* or vehicle treatment. (h) PCA of fecal metabolomes from *L. reuteri-* and vehicle-treated MS mice on P63 (*n* = 5 mice from different dams for each group). (i) Volcano plot of fecal metabolites for *L. reuteri-* and vehicle-treated MS mice (*n* = 5 mice from different dams for each group). (j) KEGG enrichment analysis for differential fecal metabolites in *L. reuteri-*treated MS mice compared with vehicle-treated MS mice. Data were shown as mean ± SEM. *P < 0.05, **P < 0.01, ***P < 0.001 (HC + vehicle vs. MS + vehicle); ^#^*P* < 0.05, ^##^*P* < 0.01, ^###^*P* < 0.001, ^####^*P* < 0.0001 (MS + vehicle vs. MS + *L. reuteri*). Statistical differences were determined by two-way ANOVA with Tukey’s multiple-comparison test (b). Statistical details are provided in Supplementary Table S1.

To examine how *L. reuteri* affected the serum levels of amino acid, we explored the effect of *L. reuteri* on the intestinal amino acid transporters of the MS mice by qPCR analyses. The expression levels of *Slc6a19* and *Slc7a8*, two lowly expressed neutral amino acid transporters in the MS mice, were increased by supplementation with *L. reuteri* (Figure 7(b)). Besides, *L. reuteri* treatment significantly increased the mRNA levels of *Slc15a1* and *Slc3a1* in MS mice. These results revealed that *L. reuteri* increases the serum amino acid levels by restoring intestinal amino acid transporter expression in the MS mice.

Since abnormal expression of intestinal amino acid transporters affects serum amino acid levels as well as fecal amino acid levels, we examined the metabolites in the feces using non-targeted metabolome analysis (Figure 7(c)). The fecal metabolomic profiles from MS mice were significantly changed compared to HC mice (Figure 7(d)). Out of 1287 fecal metabolites identified in HC mice and MS mice, 84 metabolites were found to be significantly altered by MS, of which 60 were increased, and 24 were decreased (Figure 7(e)). KEGG enrichment analysis of differential metabolites indicate that MS increased the metabolites related to amino acid metabolism in the feces (Figure 7(f)). These results suggest that low expression of intestinal amino acid transporters may lead to fewer amino acid being absorbed in the serum and more amino acid being retained in the feces of MS mice. Next, we examined the metabolites in the feces after supplementation with *L. reuteri* using non-targeted metabolome analysis (Figure 7(g)). The fecal metabolomic profiles from *L. reuteri*-treated mice were significantly changed compared to vehicle-treated controls (Figure 7(h)). Out of 1203 fecal metabolites identified in *L. reuteri*-treated mice and vehicle-treated controls, 197 metabolites were found to be significantly altered by *L. reuteri*, of which 75 were increased and 122 were decreased (Figure 7(i)). KEGG enrichment analysis of differential metabolites showed that supplementation with *L. reuteri* decreased the fecal amino acid-related metabolites. The increased fecal levels of amino acid-related metabolites in the MS mice were partially reduced by supplementation with *L. reuteri* (Figure 7(j)). These results suggest that *L. reuteri* increased the expression of intestinal amino acid transporters and led to more amino acids being absorbed in the serum and less amino acid being retained in the feces.

## Discussion

The present study reveals that changes to gut amino acid transporters and gut microbiota, particularly a decrease in the relative abundance of *L. reuteri*, are associated with MS-induced synaptic transmission deficits and behavioral abnormalities. We provide a molecular mechanism for the role of gut dysbiosis in MS-induced behavioral abnormalities, suggesting that gut dysbiosis may mediate MS-induced social deficits and anxiety by disrupting intestinal absorption of amino acid, which modulates Gln availability and maintains Glutamatergic and GABAergic function in the mPFC. Significantly, our study demonstrates that oral administration with *L. reuteri* can reverse MS-induced deficits in synaptic transmission and alleviate social deficits and anxiety-like behaviors by restoring the Gln-Glu/GABA cycle, highlighting the potential of *L. reuteri* as a therapeutic agent.

The gut microbiota is now recognized as a critical brain function and behavior regulator. Its composition can be influenced by various factors such as diet, antibiotic use, and stress.^9,11^ This study’s results align with a growing body of evidence indicating that early-life stress can cause long-lasting changes in the gut microbiota,^14,16,51^ which may contribute to the development of neuropsychiatric disorders later in life. The decrease in *L. reuteri* observed in MS mice is particularly noteworthy, as this probiotic bacterium has been previously implicated in improving social and anxiety-like behaviors in mouse models;^12,28,31,33,34^ this rescue depends upon the oxytocinergic and dopaminergic signaling in the brain,^12,31^ or through upregulation of gut microbiota-derived metabolites, such as tetrahydrobiopterin^33^ and butyrate.^28^ This suggests that microbial signals are essential in normal neurodevelopment and programming. Understanding the causal mechanisms behind this association could pave the way for next-generation microbiota-based therapeutic options (psychobiotics) for neuropsychiatric disorders like autism spectrum disorders (ASD) and stress-related conditions, including anxiety, depression, and post-traumatic stress disorder.

Studies in humans showed that abnormal brain amino acid profiles are associated with neurobehavioral deficits in ASD.^52^ Mutations in a solute carrier protein (*Slc7a5*) in the blood-brain barrier blunt the transport of branched-chain amino acids and lead to neurological and behavioral abnormalities associated with ASD in mice.^17^ Moreover, recent studies observed that the gut microbiota modulates Gln levels in the brain by regulating the expression of intestinal amino acid transporters or via modulating the levels of gut microbial-derived ammonia, which ultimately alters Gln levels in the brain and affects host behaviors, including social and depressive-like behaviors.^27,53^ We found that the levels of Gln in the blood were reduced in MS-exposed mice. Notably, abnormally low levels of Gln in the blood limit the brain’s availability. Consistent with previous studies of chronic social defeat stress-exposed and chronic forced swimming-exposed mice,^53,54^ our results demonstrate that the levels of Gln in the mPFC were reduced in MS mice. Gln is necessary for replenishing presynaptic Glu and GABA.^20^ Dysfunction in prefrontal cortical Glutamatergic and GABAergic networks has been suggested to be linked to the pathophysiology of neurobehavioral abnormalities.^55^ Our study revealed that Gln contributes to behavioral abnormalities by impairing glutamatergic and GABAergic transmission in the mPFC. Administration of *L. reuteri* reverses Gln levels in the blood and brain of MS mice. Furthermore, treatment with *L. reuteri* or Gln alleviates Glutamatergic and GABAergic dysfunction and reverses the social deficits and anxiety-like behaviors in MS mice. These interventions may work through several mechanisms, including the modulation of neurotransmitter synthesis and the restoration of normal gut microbiota function.

We observed that the levels of several amino acid-related metabolites were decreased in the serum of MS mice. One possible reason may be that the expression of amino acid transporters was impaired in the intestine. Our findings revealed that MS decreased the expression of amino acid transporters in intestinal epithelial cells. Supplementation with *L. reuteri* could restore the expression of intestinal amino acid transporters and increase Gln levels in the serum and brain of MS mice, suggesting that *L. reuteri* could directly affect amino acid absorption and ultimately improve behavioral abnormalities in MS mice. We acknowledge that the mechanism by which *L. reuteri* regulates the expression of amino acid transporters in intestinal epithelial cells is currently unknown. It is widely accepted that the gut microbiota modulates the expression of amino acid transporters in the intestinal epithelial cells through the following mechanisms: (1) metabolic byproducts from the gut microbiota, such as short-chain fatty acids (SCFAs), can influence gene expression in epithelial cells, potentially affecting the expression of amino acid transporters.^56^ (2) An imbalance in the gut microbiota may lead to intestinal inflammation, which can alter signaling pathways in epithelial cells, thereby impacting the expression and function of amino acid transporters. For example, the levels of activating transcription factor 4 (ATF4) were significantly decreased in the inflamed intestinal mucosa, and the expression of solute carrier family 1 member 5 (SLC1A5), a Gln transporter, was directly regulated by ATF4 in cell lines.^57^ (3) The gut microbiota contributes to the maintenance of the intestinal barrier, and the integrity of this barrier can indirectly affect the expression and function of amino acid transporters.^58,59^

Our findings suggest that using probiotics and nutritional interventions could be beneficial in treating stress-related disorders. The research indicates that the uptake of dietary amino acids by gut epithelial cells plays a crucial role in regulating blood and brain amino acid concentrations, which are essential for normal brain function. The imbalance of excitatory and inhibitory neurotransmitters in the brain and the activation of downstream signaling cascades may be significant factors in stress-induced neurobehavioral abnormalities. This study builds on previous research and highlights the role of gut microbiota in influencing neuropsychiatric prognosis. Furthermore, the study underscores the importance of Gln in various neurological disorders, suggesting its potential as a therapeutic target.

However, it is important to note that the study is based on a mouse model, and further research is needed to apply these findings to humans. Additionally, the study does not fully explain the molecular mechanisms through which *L.* *reuteri* and Gln affect neurotransmission and behavior. Future research should consider exploring these mechanisms in greater detail, potentially through gene expression profiling and neuroimaging techniques. Moreover, the long-term effects of *L.* *reuteri* supplementation and Gln treatment on neuropsychiatric outcomes and their potential interactions with other factors, such as diet and other gut microbiota components, should be investigated. Clinical trials involving human subjects will be essential to assess the efficacy and safety of these interventions in treating stress-related disorders, inspiring further research in this field.

In summary, our research sheds light on the communication between the microbiota, the gut, and the brain in mice with MS. We found that early life stress leads to an imbalance in the gut microbiota, which is characterized by a decreased presence of *L. reuteri*. This imbalance is followed by reduced intestinal amino acid transport and lower levels of serum Gln. The decrease in blood Gln levels limits the availability of Gln in the brain, which may contribute to the development of Glutamatergic and GABAergic deficits associated with MS. Our findings also suggest that interventions targeting the gut microbiota, such as supplementing with *L. reuteri*, may hold promise for reversing the behavioral abnormalities induced by MS. However, more research is necessary to fully comprehend the underlying mechanisms and translate these findings into clinical applications.

## Materials and methods

### Animals

Male and female C57BL/6J mice, aged 8 to 10 weeks, were procured from Chengdu ENSIWEIER Biotechnology Co., LTD. These mice were granted free access to food and water and were kept under controlled environmental conditions, including a steady ambient temperature of 22 ± 1°C and relative humidity of 50% ± 5%. The lighting regimen followed a 12-hour cycle, with illumination from 8:00 a.m. to 8:00 p.m. All procedures involving the animals were conducted in strict adherence to the guidelines of the National Institutes of Health for the care and utilization of laboratory animals and were approved by the Animal Ethics Committee of Chengdu University of Traditional Chinese Medicine.

### Early life stress

The maternal separation (MS) method was adopted with slight modifications from previously published techniques.^4^ To briefly summarize, female mice were mated and checked for seminal plugs to note the start of pregnancy (day 0.5). Pregnant female mice at 14–16 days of gestation were individually housed. From postnatal day 3 (P3) to P21, MS pups were separated from their mother and siblings for 4 hours each day. During the separation, MS pups were placed individually in a divided small chamber with clean bedding and transferred to an incubator maintained at 30 ± 1°C. The timing of the separation period was randomized but within the light cycle (9:00 a.m. to 4:00 p.m.). After separation, pups were reunited with their mother and siblings. Control pups remained undisturbed in their home cage (HC). All pups were weaned at P21 and housed in groups of three to five of the same gender until the start of the experiments. A small chamber with clean bedding and transferred to an incubator, which was maintained at 30 ± 1°C. The timing of the separation period was randomized but within the light cycle (9:00 a.m. to 4:00 p.m.). After separation, pups were reunited with the dam and littermates. Control pups remained undisturbed in the home cage (HC). All pups were weaned at P21 and housed in groups of three to five of the same gender until the start of the experiments. All tests were performed on male offspring mice.

### Three-chamber social interaction test

This test is carried out in a three-chamber box (XinRuan, XR-XJ117, Shanghai). The middle chamber is a neutral zone, while the two side chambers have wire cups or containers that can hold stimulus animals or objects. The three-chamber social interaction test was performed as previously described with minor modifications. Briefly, the test mice were adapted to the experimental room 1 hour before testing. Habituation phase: The test mouse is placed in the middle chamber and allowed to explore all three chambers freely for 10 minutes. This phase helps the mouse acclimate to the new environment. Social preference phase (sociability test): A stimulus mouse (M1), usually an unfamiliar mouse of the same species and gender, is placed in one of the side chambers, while the other side chamber contains an inanimate object (O). The test mouse is then reintroduced to the middle chamber, and its behavior is observed for 10 minutes. The time spent by the test mouse in proximity to each side chamber is recorded. A preference for the chamber with the unfamiliar mouse is considered an indication of social interest. Social memory phase (social novelty test): After the social preference phase, the test continues to assess social memory. A new unfamiliar mouse (M2) is placed in the previous inanimate object-containing chamber, and the test mouse is again allowed to explore all three chambers freely for 10 minutes. This phase assesses whether the test mice have a preference for a novel mouse over the one it has already encountered. The time spent by the test mouse in each chamber is quantified. The data is analyzed to determine the mouse’s sociability and social novelty. A mouse with normal social behavior will typically spend more time investigating the chamber with the unfamiliar mouse compared to the inanimate object-containing chamber. The time spent in each chamber is recorded using the tracking software (XinRuan, SuperMaze/VisuTrack animal behavior analysis software, Shanghai) and analyzed by independent observers. After each trial, the apparatus is cleaned to prevent any residual odors from influencing the behavior of subsequent mice.

### Open-field test

The open-field test (XinRuan, XR-XZ301, Shanghai) is a widely used behavioral assay in experimental psychology and neuroscience to assess the anxiety of rodents, typically mice or rats. A square open arena is prepared and subdivided into two zones, a center and a periphery. Firstly, the test animal is placed in the open field for 10 mins before the actual test to reduce the impact of novelty on the results. Then, the test animal is gently placed in the center of the open field. The animal’s behavior is observed for 5 mins. The movement of the animal is tracked with automated tracking systems (XinRuan, SuperMaze/VisuTrack animal behavior analysis software, Shanghai). The time spent in the center, the entries in the center, and the distance traveled in the center are measured to assess anxiety-like behavior, as rodents often prefer the periphery. After each trial, the open field is cleaned to remove any odors or waste that could affect subsequent animals.

### Elevated plus maze test

The Elevated Plus Maze (EPM, XinRuan, XR-XZ201, Shanghai) test is a widely used behavioral assay for assessing anxiety-like behavior and exploratory activity in rodents, particularly mice and rats. The EPM consists of a plus-shaped maze elevated above the floor. It has four arms, two enclosed by walls (closed arms) and two open (open arms). Firstly, the animal is allowed to acclimate to the testing room and the presence of the maze for 10 mins. Then, the test animal is placed in the maze’s center, facing one of the closed arms, to ensure unbiased starting conditions. The animal is allowed to explore the maze for 5 mins. The time spent in the open and closed arms and the number of entries into each arm are recorded using video tracking software (XinRuan, SuperMaze/VisuTrack animal behavior analysis software, Shanghai). The time spent in open arms and the number of open arms entries are often used as an index of anxiety-like behavior. The moving speed is measured to assess motor ability levels. After each trial, the apparatus is cleaned again to prevent any residual odors from affecting subsequent tests.

### Non-targeted metabolomics

Samples of serum and feces were collected and immediately frozen in liquid nitrogen, then stored at −80°C. The analysis was carried out by Novogene Co., Ltd. (Beijing, China). The samples underwent UHPLC-MS/MS analysis in positive or negative polarity mode using a Vanquish UHPLC system coupled with an Orbitrap QExactive HF-X mass spectrometer. A Hypesil Gold column was used with a 17-minute linear gradient at a flow rate of 0.2 mL/min. For positive polarity mode, eluent A (0.1% formic acid in water) and eluent B (methanol) were used, while for negative polarity mode, eluent A (5 mM ammonium acetate, pH 9.0) and eluent B (methanol) were used. Compound Discoverer 3.1 was used to process the raw data files generated by UHPLC-MS/MS, performing peak alignment, peak picking, and quantitation for each metabolite, and matching peaks with mzCloud, mzVault, and MassList databases for accurate qualitative and relative quantitative results. Metabolites were annotated using the KEGG database, HMDB database, and LIPIDMaps database. Following data preprocessing, metabolites underwent quality control, and log_2_-scaled data were used to assess the coefficient of variation in the QC samples.

### Amino acid analysis

After obtaining blood and brain tissue specimens, they were quickly frozen in liquid nitrogen. Amino acids were identified using commercially available assay kits, following the manufacturers’ guidelines. Gln in blood and brain tissues was quantified using the Gln Colorimetric Assay Kit (catalog number K556–100) from BioVision. The concentrations of Glu in the blood and brain were determined using the Glutamate ELISA Kit from Renjiebio (product code RJ17145). Additionally, the mPFC levels of GABA were measured using the mouse GABA ELISA Kit from Biorbyt (catalog number Orb782385).

### Antibiotics treatment

Vancomycin (MB1260–3), neomycin (MB1716–1), ampicillin (MB1507–2) and metronidazole (MB2200–1) were obtained from Meilunbio (Dalian, China). Broad-spectrum antibiotic treatment was performed as previously described with minor modification.^28^ Briefly, mice received a mixture of vancomycin (0.5 mg/ml), neomycin (1 mg/ml), ampicillin (1 mg/ml) and metronidazole (1 mg/ml) in drinking water from P21 to P63. Behavioral tests and fecal 16S rRNA sequencing were performed at P56-P63.

### Fecal microbiota transplantation

Feces were collected from HC mice and MS mice at P56. The freshly collected feces were diluted in saline (40 mg feces/ml saline), homogenized, and filtered with a stainless steel sieve (pore size: 0.25 mm). Fecal mixtures were mixed with 10% autoclaved glycerol and frozen at − 80°C until use. Gut microbiota-depleted mice were generated as previously described with minor modification.^28^ Briefly, the recipient HC mice were given a mixture of vancomycin (0.5 mg/ml), gentamicin (1 mg/ml), ampicillin (0.5 mg/ml), and streptomycin (1 mg/ml) in their drinking water for 7 days (P21-P27). 3 days later, the recipient animals were administered 200 µl of the fecal mixture by oral gavage once every day for 3 days. Then behavioral tests and fecal 16S rRNA sequencing for the recipient animals were performed at P56-P63.

### 16S rRNA sequencing

The fecal DNA was extracted from preserved fecal samples using the DNeasy PowerSoil Kit (Qiagen). The sequencing was carried out by Novogene Co., Ltd. in Beijing, China, using the Illumina MiSeq sequencing platform. The sequencing targeted the V3 and V4 variable regions of the 16S rRNA gene from prokaryotes and involved two rounds of paired-end reads, each covering 300 nucleotide bases. The process of clustering operational taxonomic units (OTUs) was based on a 97% similarity threshold and was facilitated by VSEARCH software version 2.4.2. One representative sequence was chosen from each cluster using the QIIME software package, version 1.8.0. Subsequently, these selected reads went through annotation and were subjected to a BLAST search against the SILVA database, version 123, using the RDP Classifier with a confidence level set at 70%. The microbial diversity in the fecal samples was assessed by calculating α-diversity metrics, specifically the count of observed species. To analyze the community structure, the UniFrac distance matrix was computed using the QIIME software and was then used in a weighted UniFrac principal coordinate analysis.

### Quantitative real-time polymerase chain reaction (qPCR) analysis

We utilized TRIzol Reagent by Invitrogen to extract total RNA from the medial prefrontal cortex (mPFC), following the manufacturer’s protocol. We then synthesized complementary DNA (cDNA) and assessed its purity and quantity using established methods. To determine if the extracted RNA is undegraded and purified, we measured the absorbance ratios at 260/280 nm. A 260/280 ratio between 1.8 and 2.1 indicates that the RNA is undegraded and purified. For qPCR, we used the StepOnePlus Real-Time PCR System from Applied Biosystems along with the SYBR Green PCR Master Mix, also from Applied Biosystems. qPCR conditions were 95°C for 30 s, followed by 40 cycles of 95°C for 5 s and 60°C for 30 s, and 72°C for 1 min. Data were analyzed using the ΔΔCt method with *Gapdh* serving as the reference housekeeping gene. The specific primer sequences employed in the study can be found in supplementary Table S2.

### Bulk rna-seq

Total RNA was extracted from the mPFC of mice using TRIzol (Invitrogen) according to the manufacturer’s instructions. There were five mice in each group. The first strand synthesis and all subsequent library preparation steps RNA was extracted from the mPFC of mice using TRIzol (Invitrogen) according to the manufacturer’s instructions. Each group consisted of five mice. Following the manufacturer’s instructions, the first strand synthesis and subsequent library preparation steps were performed using the NEBNext Ultra Directional RNA Library Prep Kit for Illumina (New England Biolabs, E7420). mRNA sequencing libraries were prepared and sequenced on the NovaSeq6000 platform (Illumina). For bulk RNA-seq data, transcripts were mapped and quantified using the HISAT2 (version 2.0.5)-StringTie (version 1.3.4) pipeline. Differential expression genes (DEGs) analysis was performed by DESeq2 (version 1.24.0) using a threshold of adjusted *p* ≤ 0.05 and |log2(cf)| ≥ 0. These DEGs were used in Gene Ontology term (GO) enrichment analysis and protein-protein interaction (PPI) networks functional enrichment analysis. The NEBNext Ultra Directional RNA Library Prep Kit for Illumina (New England Biolabs, E7420) was performed according to the manufacturer’s instructions. mRNA sequencing libraries were prepared and sequenced on the NovaSeq6000 platform (Illumina). For bulk RNA-seq data, transcripts were mapped and quantified using the HISAT2 (version 2.0.5)-StringTie (version 1.3.4) pipeline. Differential expression genes (DEGs) analysis was performed by DESeq2 (version 1.24.0) under a threshold of adjusted *p* ≤ 0.05 and |log_2_(cf)| ≥ 0, and then, these DEGs were used in Gene Ontology term (GO) enrichment analysis and protein-protein interaction (PPI) networks functional enrichment analysis.

### Culture and treatment with L. reuteri

The *L. reuteri* strain (ATCC23272) was obtained from the Beijing Biobw Biotechnology Corporation. It was cultured anaerobically in MRS medium at a constant temperature of 37°C. After cultivation, the bacterial cells underwent centrifugal sedimentation, followed by rinsing with an anaerobic phosphate-buffered saline (PBS) solution, and then were cryopreserved at −80°C for future use in experiments. The live *L.* *reuteri* was added to the drinking water, which was replaced daily to ensure a consistent dosage. On P21, the experimental group received the live bacteria, while the control group received an equivalent volume of PBS. The dose of live *L. reuteri* was set at 1 × 10^8^ CFU per mouse per day, delivered continuously through the drinking water. Throughout the experimental period, from P21 to P63, the mice had unrestricted access to the modified drinking water. The drinking water for each group was refreshed daily, two hours before the start of the dark cycle, to standardize microbial exposure and minimize variability. Behavioral assessments began after a 5-week treatment period with either *L. reuteri* or PBS. At the end of the treatment phase, on P63, fecal specimens were collected from the subjects to analyze microbial community composition using 16S rRNA gene sequencing.

### Electrophysiological recordings

The following procedure was used to record electrophysiological activity in mice. First, the mice were anesthetized with a dose of 45 mg/kg sodium pentobarbital administered intraperitoneally. Then, the animals underwent intracardiac perfusion with a cold solution composed of glucose (22.0 mM), sucrose (209.0 mM), sodium pyruvate (3.1 mM), NaHCO3 (26.0 mM), MgSO_4_•7 H_2_O (4.9 mM), sodium l-ascorbate (12.0 mM), and NaH_2_PO_4_ (1.25 mM). This solution was equilibrated with a mixture of 95% O_2_ and 5% CO_2_ to stabilize the pH between 7.2 and 7.4. The mPFC was then sectioned into 300 μm thick slices using a vibratome (Leica VT1200 S, Leica Biosystems). Subsequently, the sections were placed in artificial cerebrospinal fluid (aCSF) containing MgCl_2_ (2.0 mM), NaHPO_4_ (1.25 mM), NaCl (128.0 mM), KCl (3.0 mM), CaCl_2_ (2.0 mM), d-glucose (10.0 mM), and NaHCO_3_ (24.0 mM). The aCSF was oxygenated with the same gas mixture to maintain a pH of 7.2–7.4 and an osmolality of 295–305 mOsm. The brain slices were placed in this solution for 1 hour at a temperature of 28°C. After incubation, the slices were brought back to room temperature for whole-cell patch-clamp recordings using the pCLAMP 10 software and the MultiClamp 700B amplifier from Molecular Devices. Neurons were held at a potential of −70 mV in voltage-clamp mode to capture miniature inhibitory postsynaptic currents (mIPSCs) or excitatory postsynaptic currents (mEPSCs). For mIPSC recordings, the pipettes with a resistance of 4–6 MΩ were filled with an internal solution containing HEPES (10.0 mM), EGTA (5.0 mM), CsCl (153.3 mM), magnesium ATP (4.0 mM), and MgCl_2_•6 H_2_O (1.0 mM), adjusted to a pH of 7.25 with CsOH and an osmolality of 280–300 mOsm. Tetrodotoxin (TTX, 10 μM) and CNQX (10 μM) were present in the extracellular medium during these recordings. For mEPSC recordings, the patch electrodes were filled with an internal solution consisting of cesium gluconate (122.5 mM), Q×314(5.0 mM), EGTA (0.2 mM), magnesium ATP (4.0 mM), HEPES (10.0 mM), MgCl_2_ (1.0 mM), sodium GTP (0.3 mM), and CsCl (17.5 mM), adjusted to a pH of 7.25 with CsOH and an osmolality of 280–300 mOsm. TTX (10 μM) and bicuculline (20 μM) were added to the extracellular solution for these recordings. The recorded data were low-pass filtered within the 2–20 kHz range and digitized at a sampling rate of 5–50 kHz.

### Statistical analyses

Statistical analysis was conducted using Prism software (GraphPad). Data were evaluated for normal distribution and plotted in the figures as mean ± SEM. In each figure, n denotes the number of independent biological replicates. No samples or animals were excluded from the analyses. Differences between the two treatment groups were assessed using two-tailed, unpaired Student *t*-test. Differences among > 2 groups with only one variable were assessed using one-way ANOVA with Bonferroni post hoc test. Two-way ANOVA with Bonferroni post hoc test was used for ≥ 2 groups with two variables. Significant differences emerging from the above tests are indicated in the figures by *p < 0.05, **p < 0.01, ***p < 0.001, ****p < 0.0001.Notable non-significant differences are indicated in the figures by “ns”. Statistical significance was defined as *p* < 0.05. Exact *p* values, F values and other detailed statistical information for each figure are provided in Supplementary Table 1.

## Supplementary Material

Supplemental Material

## Data Availability

The 16S rRNA and transcriptomic sequencing datasets are available from the NCBI SRA database with the accession numbers PRJNA1158309 and PRJNA1148786. Other data that support the findings of this study are available upon reasonable request from the corresponding author Y. Yang. Source data are provided in this paper. More information on all experimental methods and data analysis are available in the Supplementary Materials and Methods.
