# Supplementary material for: Gut microbiota mediate early life stress-induced social dysfunction and anxiety-like behaviors by impairing amino acid transport at the gut
Source: Gut Microbes. 2024 Sep 11;16(1):2401939. doi: 10.1080/19490976.2024.2401939 (PMC11404583; doi:10.1080/19490976.2024.2401939)
Supplement: Supplemental Material [file KGMI_A_2401939_SM6949.zip › Supplementary information (1).docx]

Supplementary Information

Supplementary Table 2. Primers used in this study

| Gene ID | Amplification product size (bp) | Primers | Sequence (5’→3’) | Length | Tm (℃) | location |
| --- | --- | --- | --- | --- | --- | --- |
| 56643 | 173 | SLC15A1-F | CCGGCACACCCTTCTAGTG | 19 | 62.0 | 1377-1395 |
|  |  | SLC15A1-R | TGGCGTTGTGACTGGTGAC | 19 | 62.5 | 1549-1531 |
| 30962 | 172 | SLC7A9-F | GAGGAGACGGAGAGAGGATGA | 21 | 61.6 | 18-38 |
|  |  | SLC7A9-R | CCCCACGGATTCTGTGTTG | 19 | 60.4 | 189-171 |
| 74338 | 105 | SLC6A19-F | CAGGTGCTCAGGTCTTCTACT | 21 | 60.6 | 812-832 |
|  |  | SLC6A19-R | CGATCACAGAATCCATCTCACAA | 23 | 60.1 | 916-894 |
| 20510 | 176 | SLC1A1-F | CTTCCTACGGAATCACTGGCT | 21 | 61.3 | 39-59 |
|  |  | SLC1A1-R | CGATCAGCGGCAAAATGACC | 20 | 62.0 | 214-195 |
| 20538 | 227 | SLC6A20-F | CCTGCAAAACCGCCGATCTA | 20 | 62.6 | 80-99 |
|  |  | SLC6A20-R | GAGGAACAGCGTGTATGGAATC | 22 | 60.7 | 306-285 |
| 20539 | 221 | SLC7A5-F | ATATCACGCTGCTCAACGGTG | 21 | 62.8 | 149-169 |
|  |  | SLC7A5-R | CTCCAGCATGTAGGCGTAGTC | 21 | 62.0 | 369-349 |
| 17254 | 183 | SLC3A2-F | TGATGAATGCACCCTTGTACTTG | 23 | 60.8 | 857-879 |
|  |  | SLC3A2-R | GCTCCCCAGTGAAAGTGGA | 19 | 61.2 | 1039-1021 |
| 215113 | 133 | SLC43A2-F | TGCACCGCTGTGTTGGAAA | 19 | 62.7 | 49-67 |
|  |  | SLC43A2-R | CCGTGCTGTTAGTGACATTCTC | 22 | 60.9 | 181-160 |
| 20540 | 177 | SLC7A7-F | CACCACCAAGTATGAAGTGGC | 21 | 60.9 | 9-29 |
|  |  | SLC7A7-R | CCCTTAGGGGAGACAAAGATGC | 22 | 62.1 | 185-164 |
| 72472 | 147 | SLC16A10-F | GAGGTGGAGCTGACGAGGT | 19 | 63 | 139-157 |
|  |  | SLC16A10-R | CATGGACACGAAGAGCACCC | 20 | 62.8 | 285-266 |
| 50934 | 219 | SLC7A8-F | TGTGACTGAGGAACTTGTGGA | 21 | 60.7 | 756-776 |
|  |  | SLC7A8-R | GTGGACAGGGCAACAGAAATG | 21 | 61.5 | 974-954 |
| 14433 | 123 | Gapdh-F | AGGTCGGTGTGAACGGATTTG | 21 | 62.6 | 8-28 |
|  |  | Gapdh-R | TGTAGACCATGTAGTTGAGGTCA | 23 | 60.2 | 130-108 |

**
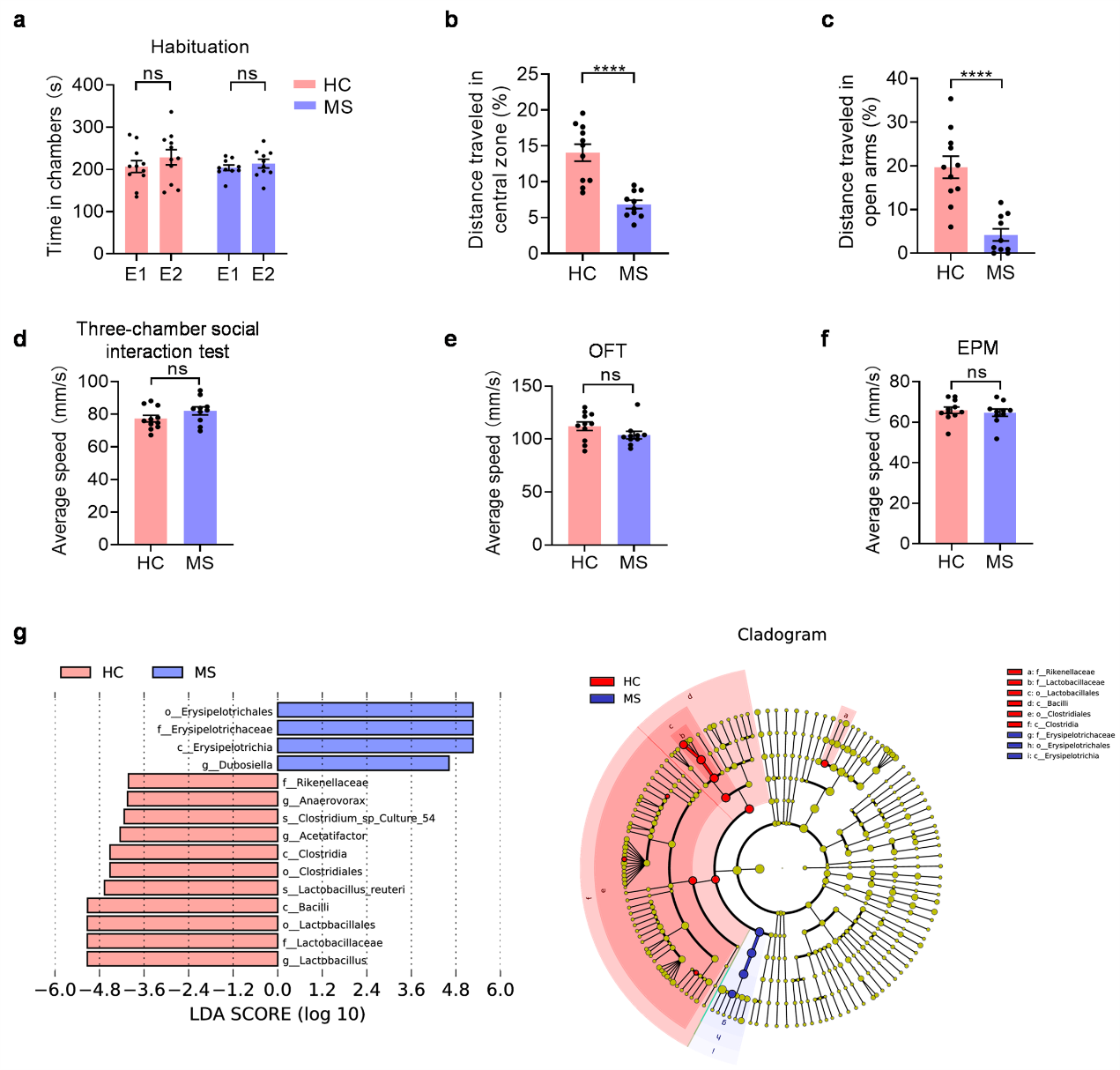
Supplementary Figure Legends**

**Supplementary Figure 1.** Behavioral dysfunction and dysbiosis of the gut microbiota in MS mice (related to Figure 1). (a) The resident time in chambers of the test mice in habituation session (n = 10-11 mice from different dams for each group). (b) The distance traveled in the central zone of the test mice in open field test (n = 10-11 mice from different dams for each group). (c) The distance traveled in the open arms of the test mice in elevated plus maze test (n = 10-11 mice from different dams for each group). (d) The average moving speed of the test mice in three-chamber social interaction test (n = 10-11 mice from different dams for each group). (e) The average moving speed of the test mice in open field test (n = 10-11 mice from different dams for each group). (f) The average moving speed of the test mice in elevated plus maze test (n = 10-11 mice from different dams for each group). (g) Bar chart showing the log-transformed LDA scores and cladogram showing the phylogenetic relationships of gut bacterial taxa found to be significantly associated with HC and MS by LEfSe (n = 5 mice from different dams for each group).


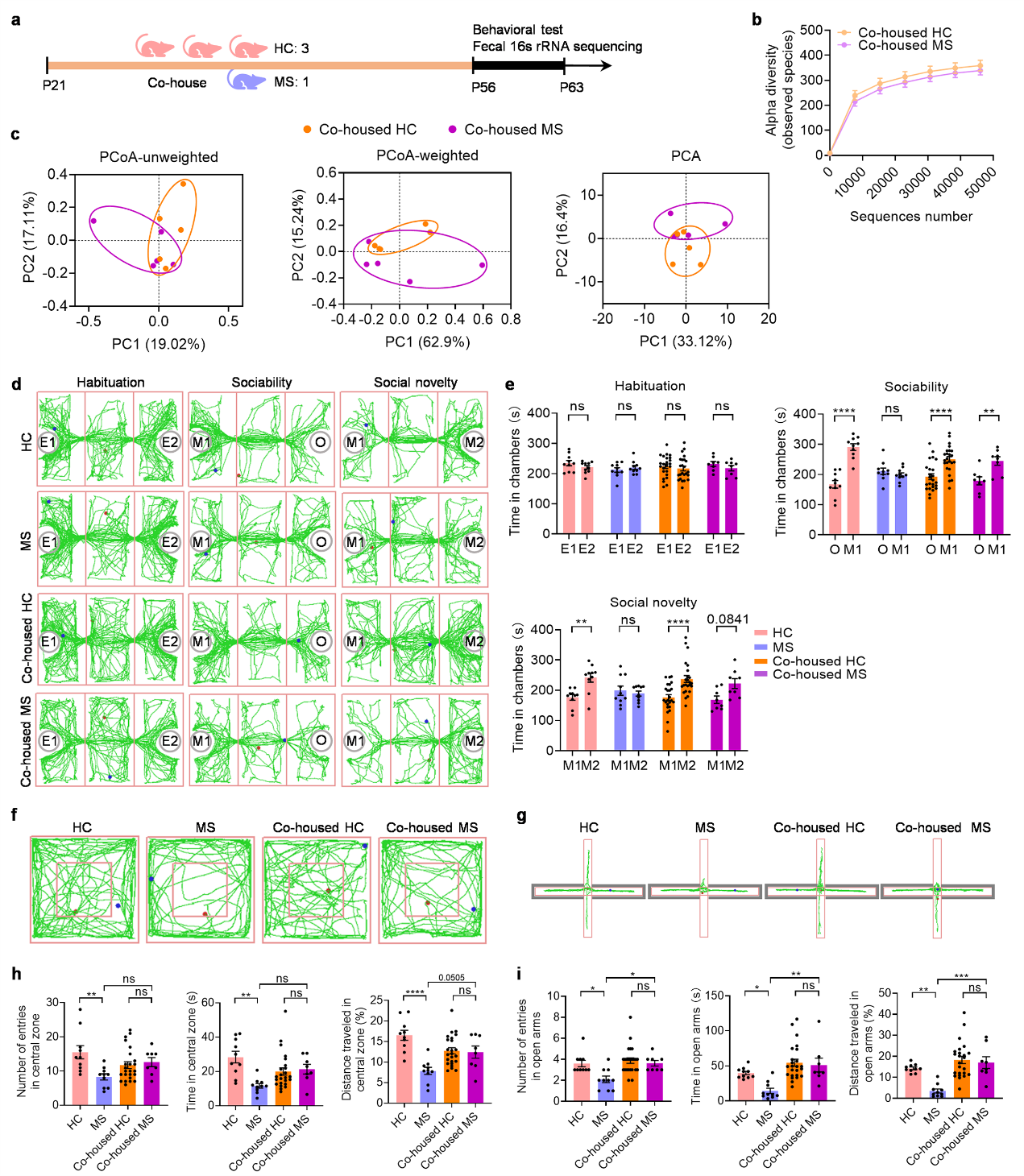


**Supplementary Figure 2.** Cohousing MS mice with HC mice rescues both social dysfunction and anxiety in MS mice. (a) Schematic of the cohousing experiment. At P21, one MS mouse was co-housed with three HC mice. (b)B α diversity of fecal 16S rDNA sequencing data from co-housed HC and MS mice. (n = 5 mice from different dams for each group). (c) PCoA of unweighted UniFrac distances (left), PCoA of weighted UniFrac distances (middle) and PCA plot (right) based on the 16S rRNA gene sequencing dataset from the feces of co-housed HC mice and co-housed MS mice. (d) Representative traces of HC mice, MS mice, co-housed HC mice and co-housed MS mice in three-chamber social interaction test. (e) The resident time in chambers of the test mice in three-chamber social interaction test (for HC, n = 10 mice from different dams; for MS, n = 10 mice from different dams; for co-housed HC, n = 24 mice from different dams; for co-housed MS, n = 8 mice from different dams). (f) Representative traces of the test mice in open field test. (g) Representative traces of test mice in the elevated plus maze test. (h) The number of entries into the center (left), the time spent in the center (middle) and the distance traveled in the central zone (right) of the test mice in open field test (for HC, n = 10 mice from different dams; for MS, n = 10 mice from different dams; for co-housed HC, n = 24 mice from different dams; for co-housed MS, n = 8 mice from different dams). (i) The number of entries into the open arms (left), the time spent in the open arms (middle) and the distance traveled in the open arms (right) of the test mice in elevated plus maze test (for HC, n = 10 mice from different dams; for MS, n = 10 mice from different dams; for co-housed HC, n = 24 mice from different dams; for co-housed MS, n = 8 mice from different dams). Data were shown as mean ± SEM. **P* < 0.05, ***P* < 0.01, ****P* < 0.001, *****P* < 0.0001; ns, no significant difference. Statistical differences were determined by one-way ANOVA with Tukey's multiple-comparison test (h, i) and two-way ANOVA with Sidak's multiple-comparison test (b, e). Statistical details are provided in Supplementary Table 1.


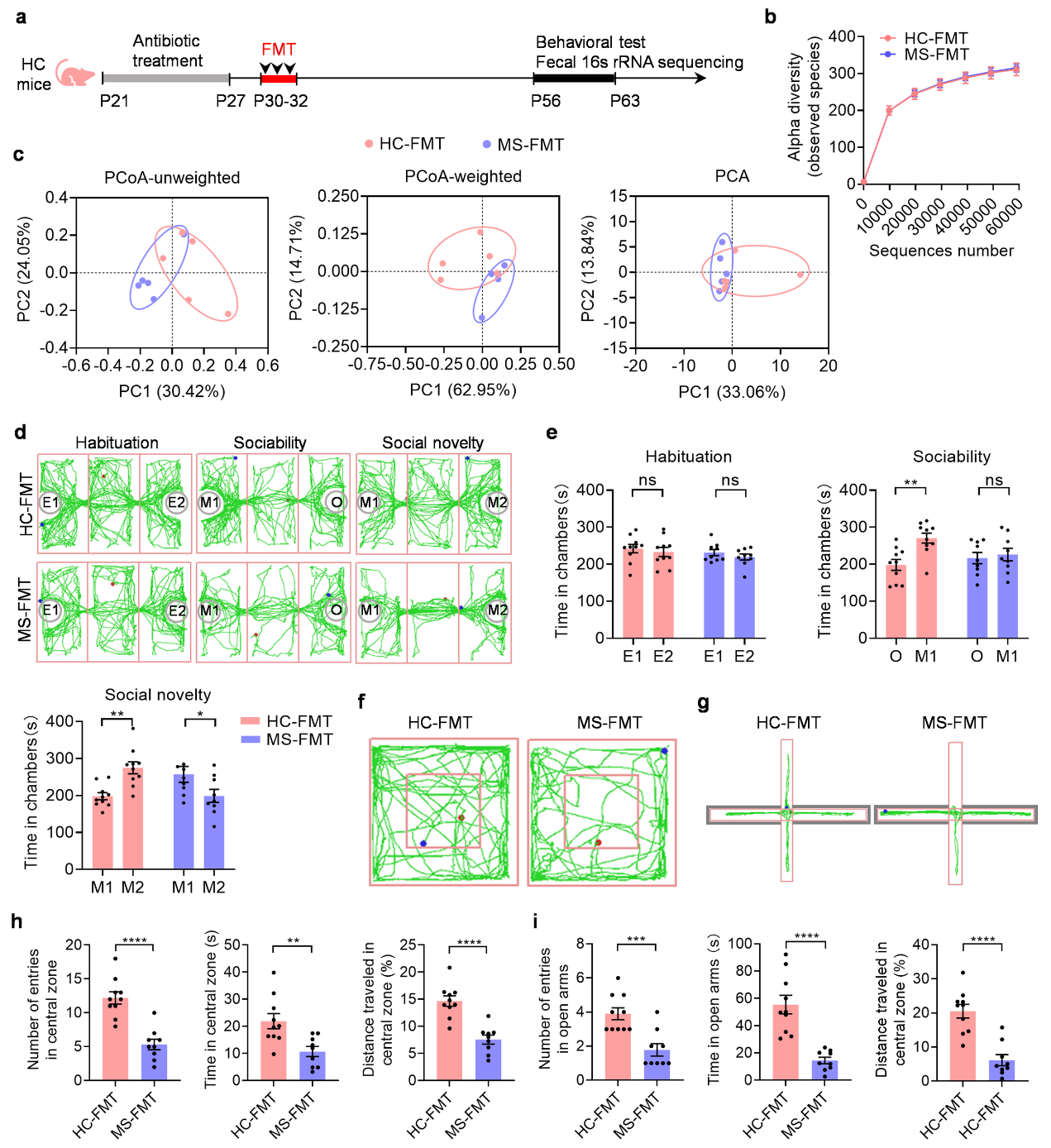


**Supplementary Figure 3.** Fecal microbiota from MS mice impairs social behavior and leads to anxiety in antibiotic-pretreated-recipient mice. (a) Schematic of the fecal microbiota transplantation (FMT) procedure. (b) α diversity of the gut microbiota of HC-FMT mice and MS-FMT mice (n = 5 mice from different dams). (c) PCoA of unweighted UniFrac distances (left), PCoA of weighted UniFrac distances (middle) and PCA plot (right) based on the 16S rRNA gene sequencing dataset from the feces of HC-FMT mice and MS-FMT mice (n = 5 mice from different dams for each group). (d) Representative traces of HC-FMT mice and MS-FMT mice in three-chamber social interaction test. (e) The resident time in chambers of the test mice in three-chamber social interaction test (n = 9-10 mice from different dams for each group). (f) Representative traces of the test mice in open field test. (g) Representative traces of the test mice in elevated plus maze test. (h) The number of entries into the center (left), the time spent in the center (middle) and the distance traveled in the central zone (right) of the test mice in open field test (n = 9-10 mice from different dams for each group). (i) The number of entries into the open arms (left), the time spent in the open arms (middle) and the distance traveled in the open arms (right) of the test mice in elevated plus maze test (n = 9-10 mice from different dams for each group). Data were shown as mean ± SEM. ***P* < 0.01, *****P* < 0.0001; ns, no significant difference. Statistical differences were determined by two-tailed unpaired Student's *t*-test (h, i) and two-way ANOVA with Sidak's multiple-comparison test (b, e). Statistical details are provided in Supplementary Table 1.


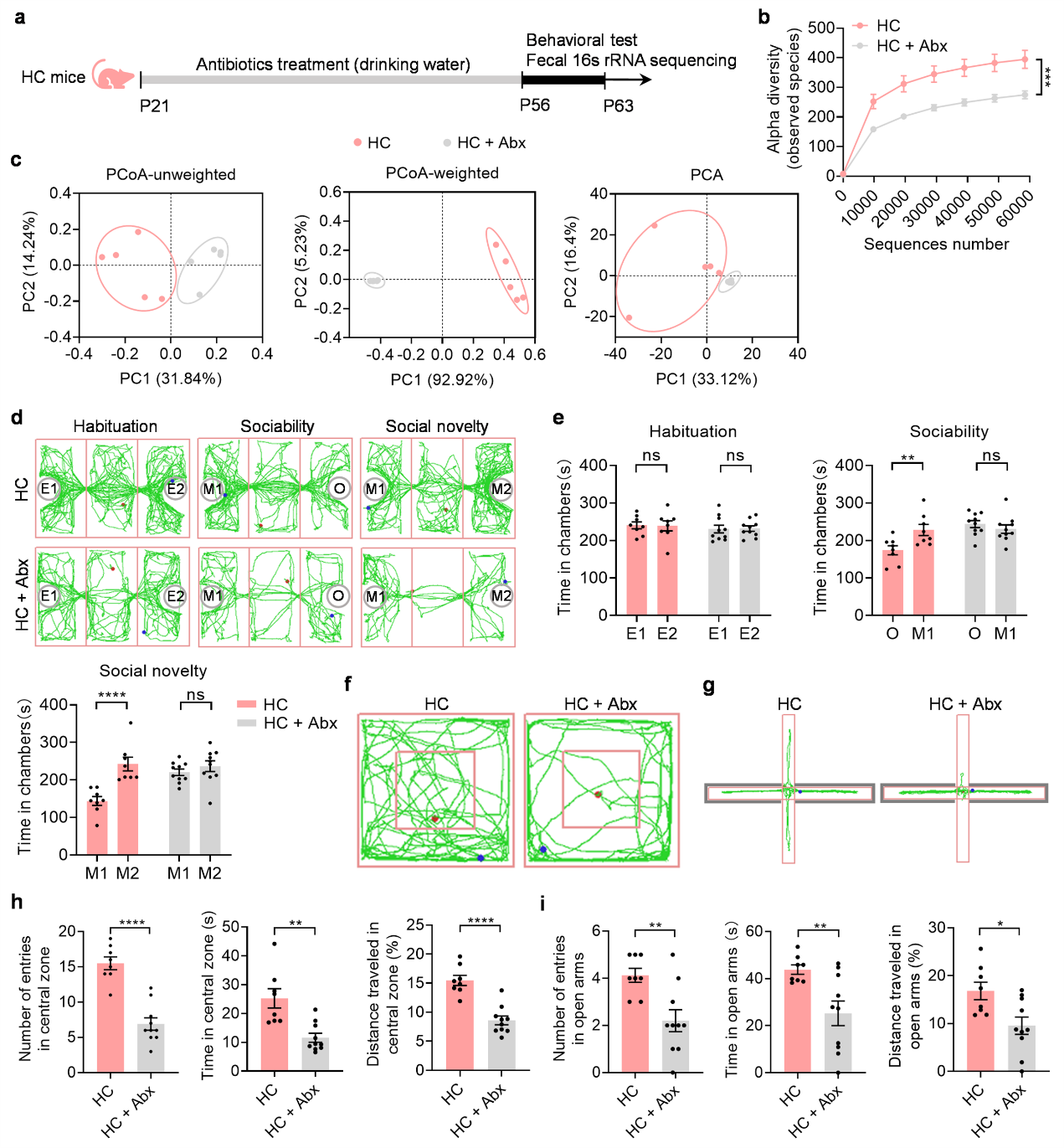


**Supplementary Figure 4.** Depletion of the gut microbiota impairs social behavior and induces anxiety in mice. (a) Schematic of depleting the gut microbiota in HC mice with antibiotics. (b) Antibiotics treatment decreased the α diversity of the gut microbiota (n = 5 mice from different dams). (c) PCoA of unweighted UniFrac distances (left), PCoA of weighted UniFrac distances (middle) and PCA plot (right) based on the 16S rRNA gene sequencing dataset from the feces of HC mice and HC + Abx mice (n = 5 mice from different dams for each group). (d) Representative traces of HC mice and HC + Abx mice in three-chamber social interaction test. (e) The resident time in chambers of the test mice in three-chamber social interaction test (n = 8-10 mice from different dams for each group). (f) Representative traces of the test mice in open field test. (g) Representative traces of the test mice in elevated plus maze test. (h) The number of entries into the center (left), the time spent in the center (middle) and the distance traveled in the central zone (right) of the test mice in open field test (n = 8-10 mice from different dams for each group). (i) The number of entries into the open arms (left), the time spent in the open arms (middle) and the distance traveled in the open arms (right) of the test mice in elevated plus maze test (n = 8-10 mice from different dams for each group). Data were shown as mean ± SEM. **P* < 0.05, ***P* < 0.01, *****P* < 0.0001; ns, no significant difference. Statistical differences were determined by two-tailed unpaired Student's *t*-test (h, i) and two-way ANOVA with Sidak's multiple-comparison test (b, e). Statistical details are provided in Supplementary Table 1.
